# Maternal and Fetal–Neonatal Complications of Romanian Women with Gestational Diabetes: A Retrospective Comparative Study

**DOI:** 10.3390/medicina61071190

**Published:** 2025-06-30

**Authors:** Adriana Gherbon, Mirela Frandes, Corina Dalia Toderescu, Darius Dirpes, Romulus Timar, Marioara Neagu Nicula, Calin Dascau, Razvan Daniluc, Bogdan Timar

**Affiliations:** 1 Department VII Internal Medicine—Diabetes, Nutrition, Metabolic Diseases, and Systemic Rheumatology, “Victor Babes” University of Medicine and Pharmacy, 300041 Timisoara, Romania; gherbon.adriana@umft.ro (A.G.); timar.romulus@umft.ro (R.T.); bogdan.timar@umft.ro (B.T.); 2 Centre of Molecular Research in Nephrology and Vascular Disease, “Victor Babes” University of Medicine and Pharmacy, 300041 Timisoara, Romania; 3 Diabetes, Nutrition, and Metabolic Diseases, “Pius Brinzeu” Emergency Hospital, 300723 Timisoara, Romania; 4 Department of Functional Sciences—Biostatistics and Medical Informatics, “Victor Babes” University of Medicine and Pharmacy, 300041 Timisoara, Romania; dariusdirpes@gmail.com; 5 Faculty of Pharmacy, “Vasile Goldis” West University, 310025 Arad, Romania; corytd11@yahoo.com; 6 Faculty of Bioengineering of Animal Resources, Discipline of Physiology, University of Life Sciences “King Mihai I”, 300645 Timisoara, Romania; marioaranicula@usvt.ro; 7 Department V Internal Medicine, “Victor Babes” University of Medicine and Pharmacy, 300041 Timisoara, Romania; dascau.calin@umft.ro; 8 Department of Internal Medicine, Railway Hospital, 300041 Timisoara, Romania; 9 Doctoral School, “Victor Babes” University of Medicine and Pharmacy, 300041 Timisoara, Romania; razvan.daniluc@umft.ro; 10 Obstetrics and Gynecology, “Pius Brinzeu” Emergency Hospital, 300723 Timisoara, Romania

**Keywords:** gestational diabetes mellitus, maternal complications, fetal complications, risk factors, obesity, chronic hypertension

## Abstract

*Background and Objectives:* Gestational diabetes mellitus (GDM) is a complex condition characterized by metabolic disorders of blood glucose that significantly impact the health of both mother and fetus. The objectives of this study were to assess the prevalence and risk factors for maternal and fetal–neonatal complications in women with GDM, comparing them to a control group (pregnant women without GDM) and pregnant women with type 1 diabetes mellitus (T1DM) or type 2 diabetes (T2DM). *Materials and Methods:* A retrospective observational study was conducted with 1418 pregnant women (279 with GDM, 74 with T1DM, 107 with T2DM, and 958 in the control group). The retrospective data included information on demographics, diagnostic test results, the medical history of pregnant women, treatments administered, identified complications, and other relevant variables for the study’s purpose. *Results:* Significant differences were found regarding maternal and neo-fetal complications between GDM and the control group in terms of abortion, pregnancy-induced hypertension, and increased fetal weight (macrosomia). Women with T1DM and T2DM showed a higher rate of abortion, premature birth, and an APGAR score of <7 at 5 min compared to those with GDM, and for T1DM, there was a higher rate of fetal mortality than in GDM cases. The primary risk factors for maternal complications included age OR = 1.03 (95% CI: 1.01–1.05, *p* = 0.002), obesity OR = 2.37 (95% CI: 1.42–3.94, *p* < 0.001), and chronic hypertension OR = 2.51 (95% CI: 1.26–5.01, *p* = 0.009). Age and obesity were also significant cofactors for maternal complications. Furthermore, the main significant risk factors for fetal–neonatal complications were obesity OR = 2.481 (95% CI:1.49–4.12, *p* < 0.001) and chronic hypertension OR = 2.813 (95% CI:1.44–5.49, *p* = 0.002), both independently and as cofactors. *Conclusions:* We found that obesity and chronic hypertension are risk factors for both maternal and fetal–neonatal complications. It is essential to prevent and adequately treat these two factors among pregnant women to avoid the onset of GDM. Additionally, screening for GDM is necessary to prevent maternal and fetal complications. Our results highlight the importance of specialized medical care and tailored management protocols in mitigating risks and ensuring positive outcomes for both mother and child during and after childbirth.

## 1. Introduction

Gestational diabetes mellitus (GDM) is a type of diabetes that develops during pregnancy in women who did not previously have diabetes. It typically occurs in the second trimester and may continue until delivery. The primary characteristic of GDM is the increase in blood glucose levels (hyperglycemia) that occurs during pregnancy, which can lead to complications for both the mother and fetus [1]. The underlying mechanism involved in the development of GDM is chronic insulin resistance. However, in 30% of cases, a deficit in insulin secretion due to beta cell dysfunction is reported [2]. According to the IDF Atlas 2025, in 2024, 23.0 million (19.7%) live births involved pregnant women with some form of hyperglycemia during pregnancy (79.2% with GDM, 11% with DM detected before pregnancy, and 9.9% with DM first identified during pregnancy). Most cases of hyperglycemia in pregnancy (89.5%) were in low- and middle-income countries, where access to antenatal care is limited. Also, regarding age, nearly half (49.2%) were women aged 45–49 years, although there are fewer pregnancies in this age group [3]. The number of GD cases is expected to rise due to the increasing prevalence of obesity [4].

DM during pregnancy is a complex condition involving metabolic disorders of blood glucose that significantly affect the health of both the mother and the fetus. This medical condition requires careful management to regulate glucose levels and prevent related complications. Therefore, it is essential to develop a comprehensive and well-structured strategy for managing gestational diabetes that addresses maternal care and promotes healthy fetal development.

Pregestational diabetes mellitus (PGDM) is a condition in which a woman has diabetes mellitus, most commonly type 1 diabetes mellitus (T1DM) or type 2 diabetes mellitus (T2DM). Before pregnancy begins, a woman is diagnosed with diabetes, increasing the risk of maternal–fetal complications. PGDM occurs in 1–2% of all pregnancies, and these rates are on the rise [5]. Approximately one in nine women (14.9 million) has DM, with 35% of those recently diagnosed being women of reproductive age [6]. This indicates that diabetes is increasingly affecting women of childbearing age. Furthermore, nearly one in five teenage girls aged 12 to 18 and one in four young adults aged 19 to 34 have prediabetes, which may progress to diabetes. The growing prevalence of diabetes among women of reproductive age is primarily due to the rising incidence of T2DM, driven by poor nutrition, obesity, and more sedentary lifestyles [7].

Several risk factors can contribute to the development of GDM. These include a personal history of gestational diabetes, a family history of diabetes, advanced maternal age, being overweight or obese, a history of metabolic syndrome, previous high birth weight babies, polycystic ovary syndrome (PCOS), recurrent spontaneous abortion, ethnic factors, and a history of cardiovascular disease [8,9].

Women undergoing in vitro fertilization (IVF) often face a higher risk of GDM due to factors such as advanced maternal age, underlying metabolic issues, and the hormonal milieu associated with fertility treatments [10]. Also, in these women, some studies show a higher risk of developing congenital heart diseases (CHDs) in offspring [11], and in case of occurrence of GDM, if poorly controlled, it may affect the activity of the reproductive axis and postnatal growth of male genital organs in the offspring [12].

The literature identifies several factors that determine maternal and fetal complications, including DM, hypertension, thyroid disorders, sexually transmitted infections, female genital tract abnormalities, substance use, exposure to teratogens, maternal age, maternal weight, multiple gestations, prior stillbirth, and prior preterm delivery [13,14].

Maternal complications encountered during pregnancy in women with GDM include pregnancy-induced hypertension (PIH), preeclampsia, infections, cesarean delivery, postpartum hypoglycemia, and a tendency for abortion. Fetal and newborn complications during pregnancy in women with gestational diabetes include macrosomia, respiratory dysfunction, neonatal hypoglycemia, and the subsequent development of diabetes [15,16].

The management of PGDM and GDM involves an integrated and careful approach aimed at ensuring a healthy pregnancy while minimizing risks to both the mother and fetus. Our study seeks to assess the prevalence and risk factors of maternal and neonatal–fetal complications that occur in women with GDM, comparing them to a control group of healthy pregnant women, as well as to those with T1DM and T2DM.

## 2. Materials and Methods

### 2.1. Study Design

The present study utilizes an observational, retrospective design. The records and medical documents of pregnant women who presented at the Timișoara Emergency County Clinical Hospital from January 2023 to December 2023 were analyzed to conduct this study. The retrospective data included information such as demographic details, the results of diagnostic tests, the medical histories of the pregnant women, the treatments administered, the complications identified, and other variables relevant to the study’s purpose.

The study was conducted in accordance with the Declaration of Helsinki guidelines, and the Ethics Committee of the “Pius Brinzeu” Emergency Hospital, Timisoara, approved the protocol.

### 2.2. Participants

**Figure 1:**
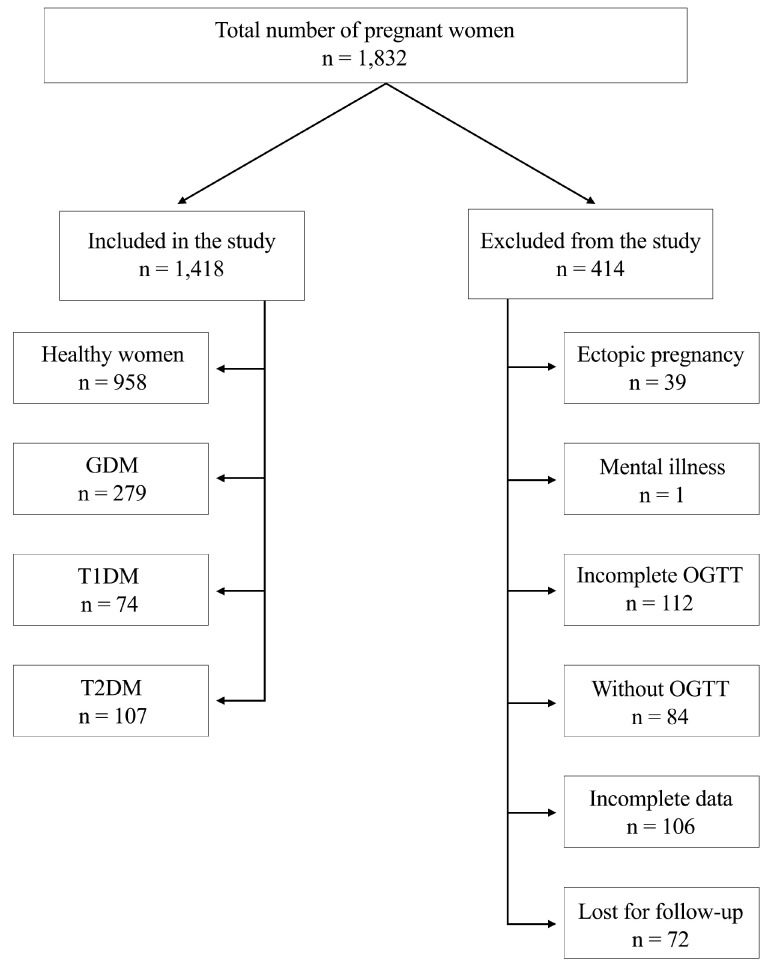
Diagram of the study flow. Abbreviations: GDM: Gestational diabetes mellitus; T1DM: Type 1 diabetes mellitus; T2DM: Type 2 diabetes mellitus; OGTT: Oral glucose tolerance test.

The participants in this study consist of 1418 pregnant women admitted to Timișoara County Emergency Clinical Hospital between January 2023 and December 2023, selected based on the study’s specific inclusion criteria. All types of pregnancies were singletons, and pregnancies resulting from IVF were not included. The inclusion criteria encompassed pregnant women diagnosed with GDM, T1DM, or T2DM, as well as pregnant women without DM (control group), all admitted to the hospital during the study period. The exclusion criteria included insufficient recorded data, refusal to participate in the study, and the presence of a mental illness. The study flowchart is presented in Figure 1.

No restrictions were applied regarding the participants’ age or other demographic characteristics. Information on participants was obtained from their medical records, including medical history, test results, and other relevant data for the study’s purpose. The demographic and clinical data of the participants were used to analyze and interpret the study results. It is essential to note that participants in this study were treated according to standard medical protocols and practices for gestational diabetes, and data analysis was conducted in accordance with the regulations and ethical guidelines of medical research. All participants and their data were treated confidentially and anonymously in the study.

### 2.3. Laboratory Characteristics

The diagnosis of GDM (according to the 10th revision of the International Classification of Diseases (ICD-10) was made with a 75 g oral glucose tolerance test (OGTT) (using venous blood samples) at 24–28 weeks of gestation [17]. The diagnosis of GDM is made when any of the following plasma glucose values are met or exceeded: Fasting: 92 mg/dL (5.1 mmol/L), 1h: 180 mg/dL (10.0 mmol/L), or 2h: 153 mg/dL (8.5 mmol/L) [18].

The level of hemoglobin A1c (HbA1c) was measured using an immune turbidimetric assay standardized by the National Glycohemoglobin Standardization Program (NGSP) and compliant with the Diabetes Control and Complications Trial (DCCT). The assay was produced by Hoffman-La Roche Ltd. in Basel, Switzerland, and has an inter-measurement coefficient of variation of 1.64% according to the manufacturer’s specifications. The reference range was 4.8–6.4% [19].

The plasma glucose level was measured using an enzyme technique with glucose oxidase. We defined normal values for fasting glucose as between 70 and 110 mg%, and for postprandial glycemia as below 140 mg%. For the diagnosis of DM, the criteria included values equal to or greater than 126 mg% for fasting glucose and 200 mg% for postprandial glycemia.

Blood pressure (BP) was measured in the left upper arm of seated subjects who had rested for 5 to 10 min. We used a conventional sphygmomanometer (Disytest, Jungingen, Germany) with an appropriate bladder size. Systolic BP (SBP) was determined as the average of two measurements taken within 10 min. According to the 2019 European Society of Cardiology guidelines, patients are classified into the following categories based on blood pressure values: ideal blood pressure (<120 systolic and <80 diastolic), normal blood pressure (120–129 systolic and/or 80–85 diastolic), normal high blood pressure (130–139 and/or 85–89 diastolic), grade I hypertension (140–159 systolic and/or 90–99 diastolic), grade II hypertension (160–179 systolic and/or 100–109 diastolic), and hypertension grade III (>180 systolic and/or 110 diastolic) [20].

Each core outcome was defined according to the proposed coding and definition, when attainable [21,22]. Maternal age at delivery was recorded in years, and pregnancy duration was documented in days based on ultrasound dating. Parity was recorded as nulliparous or multipara (more than one delivery) based on the number of previous deliveries. Body mass index (BMI) was assessed at the first prenatal visit and classified according to the World Health Organization’s definition: underweight BMI < 18.5 kg/m^2^, average weight BMI between 18.5 and 24.9 kg/m^2^, overweight BMI between 25.0 and 29.9 kg/m^2^, obese class I BMI between 30.0 and 34.9 kg/m^2^, obese class II BMI between 35.0 and 39.9 kg/m^2,^ and obese class III BMI ≥ 40.0 kg/m^2^. Chronic hypertensive disease was defined as hypertension diagnosed before pregnancy or blood pressure exceeding 140/90 mmHg before the 20th week of gestation.

Gestational hypertension is characterized as high blood pressure without proteinuria occurring after the 20th week of pregnancy on two or more occasions, spaced at least 6 h apart, in a woman who had normal blood pressure prior to 20 weeks of gestation. Preeclampsia is defined as blood pressure equal to or greater than 140/90 mmHg, accompanied by proteinuria (≥300 mg/24 h), occurring on two or more occasions at least 6 h apart, and after 20 weeks of gestation.

The statistical analysis employed a composite of neonatal and maternal outcomes. The maternal composite encompassed preeclampsia, pregnancy-induced hypertension, and emergency cesarean section. The neonatal composite comprised stillbirth, miscarriage, premature delivery, polyhydramnios, and neonatal hypoglycemia.

Preterm delivery was defined as delivery up to 37 weeks. Fetal macrosomia was described as an absolute birthweight of more than 4000g. Large for gestational age (LGA) was reported based on two different criteria: birthweight > 90th percentile and birthweight two standard deviations above the mean (both according to gestational age and sex).

Small for gestational age was reported based on two criteria: birth weight < 10th percentile and birth weight two standard deviations below the mean (according to gestational age and sex). Reference percentiles and infant size at birth were based on data from all live-born singletons without malformations within the dataset.

Intrauterine fetal death was defined according to ICD-10 codes, and perinatal mortality was defined as the number of stillbirths and deaths in the first week of life (early neonatal deaths) [23]. Asphyxia was defined as severe asphyxia, characterized by an Apgar score 0–3 at 5 min of age [24]. Miscarriage was defined as pregnancy loss before viability. Maternal infection is any infection that occurs during pregnancy, childbirth, or the postpartum period, including puerperal sepsis [25].

### 2.4. Data Collection

For the data collection in this retrospective study, the medical records of 1418 pregnant women admitted to Timișoara County Emergency Clinical Hospital were used. Relevant information was extracted from these records, including demographics, medical details, pregnancy data, clinical and laboratory results, personal medical history, and information on the treatment provided. The data collection complied with the rules and standards of medical registration, ensuring the confidentiality and anonymity of the participants.

### 2.5. Statistical Analysis

Data analysis was conducted using SPSS v.17 software (SPSS Inc., Chicago, IL, USA) and R software packages (v.3.3) for statistical computing. We regarded a *p*-value of less than 0.05 as the threshold for statistical significance, with a confidence level of 0.95 for estimating intervals. Collected data were presented as the mean (±standard deviation) for continuous variables with a Gaussian distribution, the median (inter-quartile range) for those without a Gaussian distribution, or absolute frequency (percentage) for nominal variables. The normality of continuous variable distributions was tested using the Kolmogorov–Smirnov test, while the equality of variances was assessed using Levene’s test.

The significance of the difference between groups was assessed using the Student’s *t*-test (means, Gaussian distribution), the Mann–Whitney *U* test (medians, non-Gaussian populations), and Pearson’s chi-squared or Fisher’s exact test (between proportions). The influence of one or more confounding factors on dichotomous outcomes was evaluated by deriving univariate logistic regression models. In addition, univariate and multivariate logistic regression models were employed to assess the impact of confounding factors on dichotomous outcomes. Simultaneously, the goodness of fit was calculated using Nagelkerke’s *R*^2^ method. The linearity of continuous variables in relation to the logit of the dependent variable was evaluated by applying the Box–Tidwell procedure.

## 3. Results

The study group consisted of 1418 pregnant women, including 279 with GDM, 74 with T1DM, 107 with T2DM, and 958 without DM. The average age of pregnant women with GDM was 31.78 ± 5.60 years, compared to 29.64 ± 5.91 years for the control group. The ages of the pregnant women in the study ranged from 14 to 45 years. The main characteristics of the groups are detailed in Table 1.

It was found that 62.72% of pregnant women diagnosed with GDM were primarily treated with lifestyle changes (diet, physical activity) (*p* < 0.001, X^2^ = 36.13). The study indicated that 37.28% of pregnant women with GDM also required insulin.

Notably, the age group of pregnant women aged 31–35 is the most represented in cases of GDM (31.55%) (Table 2).

Paradoxically, pregnant women considered to be at high obstetric risk (over 35 years) are also numerous (27.24%). Additionally, if we include women aged 31 to 35 years in this group, they represent 58.79% of all pregnant women. This percentage accounts for more than half of pregnant women with GDM. Our results indicate that the age groups most affected by GDM in pregnancy are those over 35 years and those between 30 and 35 years. These findings highlight that the risk of developing GDM during pregnancy increases with age, making it crucial for women in these categories to be monitored and managed appropriately. Women with GDM were older than the control group, but their ages were similar to those of T1DM and T2DM patients.

Regarding chronic diseases, the prevalence of chronic hypertension and obesity was higher than that of the control group, but not in the groups with T1DM and T2DM. Maternal and fetal–neonatal complications encountered in our study are detailed in Table 3.

The results show that regarding the type of birth for the analyzed pregnant women, 54.48% of the births were via cesarean section, while 45.52% were through natural birth (Table 3) (*p* = 0.034, X^2^ = 4.48). These figures indicate a significant prevalence of cesarean births compared to natural births among the pregnant women studied. The distribution of 54.48% for cesarean sections and 45.52% for natural births suggests that surgical interventions are more frequently utilized in this population, whether for medical reasons, personal preferences, or specific medical recommendations. Conversely, natural birth constitutes a smaller percentage compared to cesarean sections. This may indicate a relatively high prevalence of surgery in pregnant women with GDM, possibly justified by the need to manage risks and protect the health of both mother and child. We found a significant difference in cesarean section rates compared to other groups, since in Romania, this type of intervention can be performed upon request.

Pregnancy-induced hypertension (PIH) occurs in 41.93% of cases, indicating that it is a common complication associated with diabetes mellitus during pregnancy. PIH can have severe consequences for both the mother and fetus, necessitating careful monitoring and appropriate management. This complication is significantly more prevalent than in other groups.

Only 8.24% of cases showed preeclampsia, which can endanger the health of both the mother and the fetus and require close monitoring and appropriate management. It is essential to pay attention to these complications, as they can impact the long-term health of both the mother and the baby. The incidence of preeclampsia was significantly higher than in the control group, although this was not the case when compared to the T1DM and T2DM groups.

The incidence of abortion in women with GDM was higher compared to the control group; additionally, there were significant differences when compared to those with T1DM and T2DM, and the incidences were higher in the pregestational DM. We have no record of more premature births than in healthy women. Premature births were recorded more frequently in T1DM and T2DM than in GDM. The risk of hypoglycemia was similar across all groups, and we determined that there was a higher risk of infection compared to the control group.

An APGAR score of less than seven may indicate moderate adaptation difficulties in the newborn, necessitating more intensive medical attention and intervention to ensure stable health (Table 3). The APGAR scores were lower in women with T1DM and T2DM compared to those with GDM, with no differences observed between the GDM group and the control group. Our results showed that most newborns achieved high APGAR scores, particularly 9 and 10, which indicate excellent adaptation to extrauterine life.

Regarding birth weight, 11.11% had a weight of less than 2500g. According to the study, about 13.26% of newborns had birth weights exceeding 4000g. We did not find significant differences in insufficient weight gain at birth across all groups. However, for newborns with macrosomia, the prevalence was higher than in the control group, but not comparable to T1DM and T2DM. Fetal death was more common in T1DM than in GDM, but there were no differences between GDM and the other groups. The main causes of fetal death were preeclampsia, maternal infections with COVID-19, and placenta previa. We also investigated several predictors of GDM, including older age (≥35 years), the number of pregnancies, the presence of maternal obesity, chronic hypertension, anemia, and autoimmune thyroiditis (Table 4).

**Figure 2:**
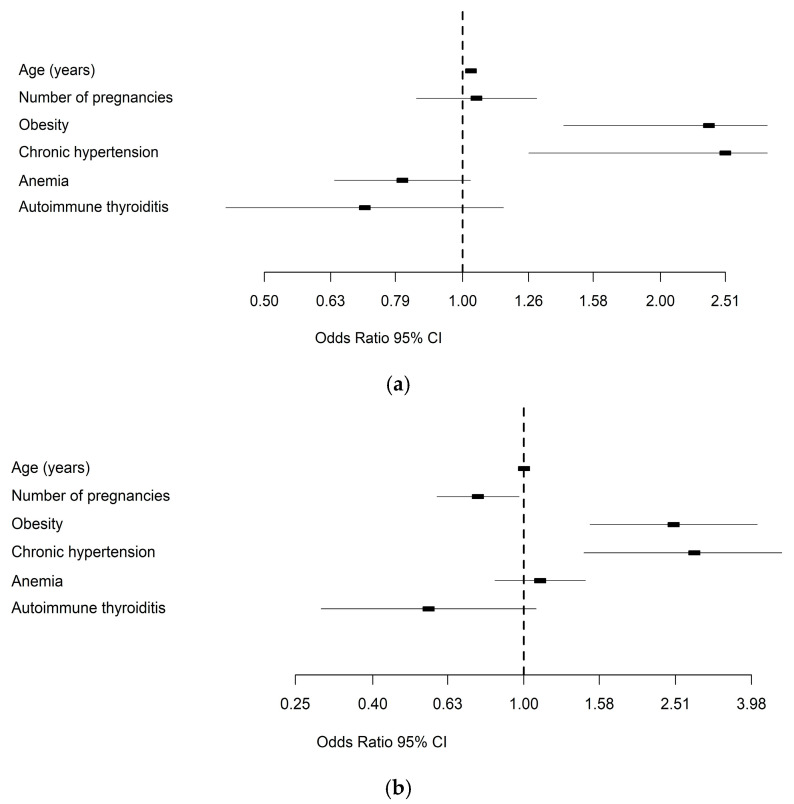
Risk factors for maternal complications (**a**) and fetal–neonatal complications (**b**).

Our study’s predictors included older age, obesity, and chronic hypertension for maternal complications, as well as obesity and chronic hypertension for fetal complications (Figure 2a). Age was identified as a risk factor for developing maternal complications (OR = 1.03, 95% CI: 1.011–1.047, *p* = 0.002). However, we found that the number of pregnancies was not a significant risk factor (OR = 1.05, 95% CI: 0.849–1.294, *p* = 0.663). Additionally, obesity proved to be a significant risk factor for maternal complications (OR = 2.37; 95% CI: 1.424–3.942; *p* < 0.001). Furthermore, chronic hypertension was also a significant risk factor for maternal complications (OR = 2.51; 95% CI: 1.262–5.002; *p* = 0.009). Conversely, age was not a risk factor for developing fetal–neonatal complications (OR = 1.001, 95% CI: 0.98–1.022, *p* = 0.942) (Figure 2b). We also noted that the number of pregnancies was not a significant risk factor (OR = 0.757, 95% CI: 0.59–0.971, *p* = 0.028). In contrast, obesity was a significant risk factor for developing fetal–neonatal complications (OR = 2.481; 95% CI: 1.493–4.12; *p* < 0.001). Moreover, chronic hypertension also emerged as a significant risk factor (OR = 2.813; 95% CI: 1.44–5.495; *p* = 0.002).

Multivariate logistic regressions were conducted to determine the influence of predictors on the likelihood of maternal and fetal–neonatal complications. The resulting regression model was statistically significant, *χ*^2^ (1411) = 27.851, *p* < 0.001, explaining 52.6% (Nagelkerke *R*^2^) of the variance in maternal complications. We found that age and obesity were statistically significant predictor variables. Older age was a risk factor (OR = 1.023, 95% CI: 1.005–1.043). The presence of obesity was associated with an increased likelihood of developing maternal complications (OR = 1.995, 95% CI: 1.169–3.405). The regression model for fetal–neonatal complications was statistically significant, *χ*^2^ (1411) = 26.305, *p* < 0.001, with a Nagelkerke *R*^2^ of 0.528. The presence of obesity resulted in 2.024 times higher odds of developing fetal–neonatal complications (OR = 2.024, 95% CI: 1.176–3.484). Additionally, the presence of chronic hypertension resulted in 2.369 times higher odds of developing fetal–neonatal complications (OR = 2.369, 95% CI: 1.173–4.785).

## 4. Discussion

Our study aims to evaluate the prevalence and risk factors of maternal–fetal complications in women with GDM, comparing these factors with a control group as well as with T1DM and T2DM. The age group of pregnant women over 35 has the highest prevalence of DM in pregnancy. This result confirms that advanced maternal age is a significant risk factor for the development of DM during pregnancy. Women who fall into this category must be adequately monitored and managed to reduce the risks associated with DM in pregnancy. Pregnant women over 35 have a higher risk of developing GDM compared to younger women. This can be attributed to age and other risk factors associated with the older age group [26,27,28]. An increased prevalence of GDM in pregnancy is observed, representing 15.22% of all cases. The main factors involved in the increasing prevalence of GDM are sedentary lifestyles, obesity among women of reproductive age, and urbanization [26]. This result emphasizes the importance of proper monitoring and management of GDM during pregnancy, as this condition can have negative consequences for both mother and fetus. Consistent with the results of our study, Fong et al. reported a higher percentage of cases with GDM (85%) compared to the remaining 15% attributed to T1 or T2 pre-existing DM [29,30].

The distribution of birth types reflects a predominance of cesarean deliveries, accounting for 54.48% of all cases. Several factors, including medical indications, patient preferences, and institutional policies, may contribute to this high percentage. However, the choice of delivery method must be individualized, considering the safety of both mother and child. Additionally, many studies have reported increased cesarean section rates in patients with GDM [31,32,33].

APGAR scores reflect the health status of newborns immediately after birth. In our study, most neonates achieved an APGAR score of 9, indicating good adaptation to extrauterine life. However, we noted a few cases with lower APGAR scores (8 and 7), which may indicate the need for additional surveillance and intervention to ensure the newborn’s stability and health. Gualdani et al. reported low APGAR scores (<7), particularly in pregnant women with T1 pregestational DM, compared to pregnant women who had pregestational T1DM and GDM [34].

In general, studies in the literature indicate that women with GDM have an increased risk of delivering newborns with high birth weight (fetal macrosomia), which can lead to complications and risks for both the mother and baby. Moreover, Ye et al. found that the odds of macrosomia rise in cases of GDM in studies where no insulin was used [16,35]. Regarding diseases associated with DM in pregnancy, there is a significant prevalence of pregnancy-induced hypertension (PIH), with 41.93% of recorded cases. This underscores the importance of monitoring blood pressure during pregnancy in women with DM, as PIH can be linked to severe complications for both the mother and fetus [36].

Regarding fetal death, the literature indicates that it is more common in T1DM. In our study, fetal death rates were higher in T1DM compared to GDM, but not when comparing GDM to other groups [37]. Fetal death can result from a variety of maternal, fetal, placental, and environmental factors. In some cases, causes can be identified, while in others, they remain unexplained. Concerning adverse maternal outcomes, other authors have shown that women with GDM had higher chances of preeclampsia, induction of labor, and cesarean section, in line with prior studies [38]. Additionally, Ye et al. found a 1.24–1.46 times higher probability of preeclampsia among 37 patients with and without GDM, consistent with previous results [16]. We did not identify complications such as shoulder dystocia or birth injuries. Neonates were monitored for hypoglycemia by a neonatologist, and none developed hypoglycemia.

The predominant treatment for GDM during pregnancy is diet, which addresses 62.72% of cases. However, around 37.28% of DM cases necessitate both diet and insulin administration. This highlights the need for an individualized approach, customized to each woman’s specific needs and treatment response. Insulin is the standard treatment for managing GDM when adequate glucose levels are not maintained through diet and exercise [39].

A treatment used to improve maternal metabolic health and thereby positively influence neonatal outcomes includes the use of nutraceuticals. They improve glycemic control, reduce oxidative stress and inflammation, and enhance placental and fetal development. Supplements such as myo-inositol, vitamin D, omega-3 fatty acids, magnesium, and antioxidants (e.g., vitamins C and E) have been shown to enhance insulin sensitivity and reduce inflammation [40,41]. Myo-inositol has demonstrated benefits in reducing the incidence of GDM in high-risk women, and vitamin D status is linked to improved placental function and a reduced risk of GDM [42,43].

Given the increased risk of GDM and potential metabolic and developmental complications in infants born after IVF, early identification and management of neonatal hypoglycemia and other metabolic disturbances are critical to preventing long-term neurodevelopmental impairments. Monitoring growth patterns, feeding adequacy, and developmental milestones ensures timely interventions [44]. Nutraceutical supplementation in IVF patients at risk of GDM provides a promising adjunct to enhance maternal metabolic health and reduce adverse neonatal outcomes. Coupled with vigilant neonatal follow-up, this approach enables early detection and management of complications, thereby optimizing both short- and long-term child health. Additionally, in autoimmune DM (such as T1DM), cell and gene therapies hold significant potential for treating these conditions, although further research is still required [45].

Clinically, pre-pregnancy obesity is a significant predictor of GDM, as body fat percentage is proportional to insulin resistance [28]. Pre-pregnancy overweight is an essential determinant of GDM, and interventions should start from the pre-pregnancy period for successful prevention of GDM [46]. The results obtained in this study provide insight into the distribution and association of essential variables in the management of pregnancy in women with DM. A multidisciplinary approach, combined with appropriate antenatal care, is necessary to monitor and manage these variables individually, ensuring a healthy course of pregnancy and delivery.

The study’s limitations include its retrospective design and small sample size. The generalizability of the findings is limited. Data collection was conducted in one public hospital, which may not entirely represent other centers in Romania. The study was conducted within a specific healthcare setting (e.g., a single hospital, region, or population group), which may not reflect broader demographic or clinical populations. For example, the sample may have specific ethnic, socioeconomic, or risk profile characteristics that are not representative of the general pregnant population. As a result, the findings may not apply to other settings with different healthcare practices, patient populations, or GDM screening protocols. This study did not follow up with women with positive GDM to explore their pregnancy outcomes and plasma glucose levels during delivery and the postpartum period. The retrospective design inherently limits the ability to establish causal relationships. Important variables such as lifestyle factors, socioeconomic status, or pre-pregnancy health behaviors may not have been recorded or uniformly available, potentially leading to unmeasured confounding. Additionally, because the study relied on secondary data, there was limited control over the timing and methods of data collection, including diagnostic criteria or testing protocols, which may have varied over time or between providers. Future research employing prospective designs, standardized diagnostic criteria, and more diverse populations is recommended to validate these findings and enhance their applicability to broader clinical and health contexts.

## 5. Conclusions

Our study shows that obesity and chronic hypertension are risk factors for both maternal and fetal–neonatal complications. It is essential to prevent and adequately treat these two factors among pregnant women to prevent the occurrence of GDM. Additionally, it is necessary to screen for GDM to prevent the occurrence of maternal and fetal complications. Moreover, our results highlight the complexity and importance of managing DM during pregnancy, as well as the necessity for meticulous monitoring and targeted interventions to ensure the health of both mother and child before, during, and after birth.

## Figures and Tables

**Table 1:** Characteristics of the studied groups and comparison between GDM and the control group, as well as T1DM and T2DM.

Parameters	GDM	Control Group	*p*-Value (GDM vs. Control Group)	T1DM	*p*-Value (GDM vs. T1DM)	T2DM	*p*-Value (GDM vs. T2DM)
Total	279	958		74		107	
Age (years)	31.78 ± 5.60	29.64 ± 5.91	<0.001	31.08 ± 6.14	0.308	33.18 ± 6.24	0.048
Areas			0.239		0.140		0.152
rural	140 (50.18%)	519 (54.17%)		30 (40.54%)		45 (42.05%)	
urban	139 (49.82%)	439 (45.83%)		44 (59.46%)		62 (57.95%)	
Number of pregnancies			0.342		0.0001		0.990
primiparous	138 (49.46%)	443 (46.24%)		56 (75.67%)		53 (49.53%)	
multiparous	141 (50.54%)	515 (53.76%)		18 (24.33%)		54 (50.47%)	
Glycemia (mg/dL)	98.97 ± 13.63	76.92 ± 8.62	<0.001	180.63 ± 87.67	<0.001	146.52 ± 65.99	<0.001
HbA1c (%)	5.45 ± 0.54	4.57 ± 0.33	<0.001	8.23 ± 2.44	<0.001	7.18 ± 2.00	<0.001
Treatment			<0.001		<0.001		<0.001
insulin	104 (37.28%)	0 (0%)		74 (100%)		93 (86.91%)	
diet	175 (62.72%)	958 (100%)		0 (0%)		14 (13.09%)	
Chronic hypertension			<0.001		0.300		<0.001
yes	11 (3.94%)	4 (0.42%)		5 (6.76%)		17 (15.89%)	
no	268 (96.06%)	954 (99.58%)		69 (93.24%)		90 (84.11%)	
Obesity			<0.001		0.584		<0.001
yes	21 (7.53%)	6 (0.63%)		7 (9.46%)		32 (29.91%)	
no	258 (92.47%)	952 (99.37%)		67 (90.54%)		75 (70.09%)	
Anemia			0.003		0.238		0.051
yes	61 (21.86%)	296 (30.90%)		21 (28.38%)		14 (13.08%)	
no	218 (78.14%)	662 (69.10%)		53 (71.62%)		93 (86.92%)	
Autoimmune thyroiditis			0.145		<0.001		0.094
yes	8 (2.87%)	47 (4.9%)		15 (20.27%)		7 (6.54%)	
no	271 (97.13%)	911 (95.1%)		59 (79.73%)		100 (93.46%)	

Notes: Continuous variables (with non-Gaussian distribution) are indicated by their median (IQR). Categorical variables are presented by absolute frequency (percentage) in the sample. Continuous variables (with Gaussian distribution) are indicated by their mean (SD). Abbreviations: GDM: Gestational diabetes mellitus; T1DM: Type 1 diabetes mellitus; T2DM: Type 2 diabetes mellitus.

**Table 2:** Distribution of cases by age groups.

Age Group	GDM (*n* = 279)	Control Group (*n* = 958)	*p*-Value
14–20 years	9 (3.22%)	67 (7%)	0.021
21–25 years	27 (9.67%)	169 (17.64%)	0.136
26–30 years	79 (28.31%)	296 (30.90%)	0.408
31–35 years	88 (31.55%)	261 (27.24%)	0.160
36–40 years	65 (23.30%)	141 (14.71%)	0.0007
41–45 years	11 (3.95%)	24 (2.5%)	0.202

Abbreviations: GDM: Gestational diabetes mellitus.

**Table 3:** Maternal–fetal complications in the studied group, comparison between GDM and the control group, and T1DM and T2DM.

Maternal and Fetal–Neonatal Complications	GDM (*n* = 279)	Control Group (*n* = 958)	*p*-Value (GDM vs. Control Group)	T1DM (*n* = 74)	*p*-Value (GDM vs. T1DM)	T2DM (*n* = 107)	*p*-Value (GDM vs. T2DM)
Maternal complications							
Type of birth			0.0035		0.0001		0.043
caesarean	152 (54.48%)	427 (44.57%)		22 (29.72%)		46 (43%)	
spontaneous	127 (45.52%)	531 (55.43%)		52 (70.27%)		61 (57%)	
Abortion			0.008		0.030		0.031
yes	2 (0.72%)	0 (0%)		3 (4.05%)		4 (3.74%)	
no	277 (99.28%)	958 (100%)		71 (95.95%)		103 (96.26%)	
Premature birth			0.088		0.004		0.014
premature	43 (15.41%)	111 (11.59%)		22 (29.73%)		28 (26.17%)	
on time	236 (84.59%)	847 (88.41%)		52 (70.27%)		79 (73.83%)	
Pregnancy-induced hypertension (PIH)			<0.001		<0.001		0.007
yes	117 (41.93%)	35 (3.65%)		12 (16.22%)		29 (27.1%)	
no	162 (58.07%)	923 (96.35%)		62 (83.78%)		78 (72.9%)	
Preeclampsia			<0.001		0.487		0.576
yes	23 (8.24%)	5 (0.52%)		8 (10.81%)		7 (6.54%)	
no	256 (91.76%)	953 (99.48%)		66 (89.19%)		100 (93.46%)	
Hypoglycemia			0.106		0.841		0.902
yes	3 (1.07%)	3 (0.31%)		1 (1.35%)		1 (0.93%)	
no	276 (98.93%)	955 (99.69%)		73 (98.65%)		106 (99.07%)	
Infections			<0.001		0.136		0.475
yes	11 (3.94%)	6 (0.63%)		6 (8.1%)		6 (5.6%)	
no	268 (96.06%)	952 (99.37%)		68 (91.9%)		101 (94.4%)	
Fetal–neonatal complications							
Apgar score			0.668		0.016	2	0.002
<7	28 (10.03%)	88 (9.18%)		15 (20.27%)		3 (21.5%)	
>7	251 (89.97%)	870 (90.82%)		59 (79.73%)		84 (78.5%)	
Birth weight							
<2500 g	31 (11.11%)	112 (11.7%)	0.789	12 (16.22%)	0.232	18 (16.82%)	0.131
2500–4000 g	211 (75.63%)	799 (83.4%)		50 (67.56%)		69 (64.48%)	
>4000 g	37 (13.26%)	47 (4.9%)	<0.001	12 (16.22%)	0.513	20 (18.7%)	0.178
Fetal death			0.706		0.017		0.079
yes	3 (1.07%)	8 (0.83%)		4 (5.4%)		4 (3.74%)	
no	276 (98.93%)	950 (99.17%)		70 (94.6%)		103 (96.26%)	

Notes: Continuous variables (with non-Gaussian distribution) are indicated by their median (IQR). Categorical variables are presented by absolute frequency (percentage) in the sample. Continuous variables (with Gaussian distribution) are indicated by their mean (SD). Abbreviations: GDM: Gestational diabetes mellitus; T1DM: Type 1 diabetes mellitus; T2DM: Type 2 diabetes mellitus.

**Table 4:** Risk factors for maternal complications and fetal–neonatal complications.

	Maternal Complications				Fetal–Neonatal Complications			
Predictor	OR	Lower	Upper	*p*-Value	OR	Lower	Upper	*p*-Value
Age (years)	1.03	1.011	1.047	0.002	1.001	0.98	1.022	0.942
Number of pregnancies	1.05	0.849	1.294	0.663	0.757	0.59	0.971	0.028
Obesity	2.37	1.424	3.942	<0.001	2.481	1.493	4.12	<0.001
Chronic hypertension	2.51	1.262	5.002	0.009	2.813	1.44	5.495	0.002
Anemia	0.81	0.64	1.031	0.088	1.104	0.839	1.453	0.48
Autoimmune thyroiditis	0.71	0.436	1.152	0.165	0.561	0.292	1.076	0.082

## Data Availability

The datasets used and/or analyzed during the current study are available from the corresponding author upon reasonable request.

## Abbreviations

GDM	Gestational diabetes mellitus
T1DM	Type 1 diabetes mellitus
T2DM	Type 2 diabetes mellitus
DM	Diabetes mellitus
PCOS	Polycystic ovary syndrome
PIH	Pregnancy-induced hypertension
ICD-10	International Classification of Diseases
OGTT	Oral glucose tolerance test
BMI	Body mass index
HELLP	Hemolysis, elevated liver enzymes, and low platelets
LGA	Large for gestational age
